# Non-invasive prenatal testing: when results suggests maternal cancer

**DOI:** 10.1515/medgen-2023-2055

**Published:** 2023-12-05

**Authors:** Liesbeth Lenaerts, Miel Theunis, Frédéric Amant, Joris R. Vermeesch

**Affiliations:** Catholic University Leuven Department of Oncology, Laboratory of Gynecological Oncology Herestraat 49 – box 818 3000 Leuven Belgium; Catholic University Leuven Centre of Human Genetics Herestraat 49 – box 818 3000 Leuven Belgium; Catholic University Leuven Department of Oncology, Laboratory for Gynecological Oncology Leuven Belgium; University Hospitals Leuven Centre of Human Genetics Leuven Belgium

**Keywords:** Cell-free DNA, non-invasive prenatal testing, incidental finding, maternal malignancy

## Abstract

It is now well-established that non-invasive prenatal testing (NIPT), originally designed to screen cell-free DNA (cfDNA) in maternal blood for the presence of common fetal trisomies, can lead to incidental detection of occult maternal malignancies. Retrospective evaluations have demonstrated that the detection of multiple copy number alterations in cfDNA is particularly suggestive of an incipient tumor and that cancer detection rates not only depend on tumor biology but also on applied NIPT technologies and downstream diagnostic investigations. Since the identification of a maternal cancer in pregnancy has implications for both woman and the unborn child, prospective studies are needed to provide evidence on best clinical practices and on clinical utility in terms of patient outcomes.

## Abbreviations

cfDNA cell-free DNA
CNA copy number alteration
ctDNA circulating tumor DNA
MCED multi-cancer early detection
NIPT non-invasive prenatal testing
WB-DWI MRI whole-body diffusion-weighted MRI

## Introduction

The identification of placenta-derived cell-free DNA (cfDNA) in the blood circulatory system of pregnant women, spurred the development of non-invasive prenatal testing (NIPT) [1]. Placental‐derived cfDNA fragments can enter the maternal bloodstream via apoptosis or necrosis of trophoblasts, where they mix with cfDNA species of maternal origin, the latter mainly originating from normal cells of the hematopoietic lineage [2]. Most NIPT methods rely on either whole-genome or targeted sequence analysis of the mixture of cfDNA fragments in maternal plasma to screen for the presence of common fetal trisomies 21, 18 and 13 [3]. Since placental-derived cfDNA species exist in a high background of maternal cfDNA*,* NIPT findings can be confounded by maternal (segmental) chromosomal imbalances [4]. The latter can be constitutional mosaicisms or acquired chromosomal aberrations originating from a maternal neoplasia. Indeed, malignant as well as benign tumors can shed cell-free tumor DNA (ctDNA) fragments into the blood circulatory system [5]. It is now evident that NIPT has the potential to identify tumor-specific copy number alterations (CNAs), comprising segmental and/or whole chromosome aneuploidies, and, as a consequence, can indirectly detect (incipient) maternal cancers [3]. As the use of NIPT is increasingly expanding globally for pregnancies with advancing maternal age and its scope is being broadened beyond aneuploidy screening, more aberrant test results caused by a maternal cancer are expected to come to the foreground. Furthermore, whereas most European countries currently offer NIPT as a contingency test only in high-risk pregnant women [5], [6], continuously decreasing costs of NIPT are expected to stimulate changing policies towards implementation of NIPT as a prenatal screening test in a broader population. This has recently also been recommended by ACMG evidence-based guidelines [6]. At present, in Europe, NIPT as a first tier test for the general pregnant population is only implemented in Belgium and the Netherlands [7], [8].

Given that malignancies co-occurring with a pregnancy are rare, affecting about 1 in 1000 to 2000 pregnancies [9], a NIPT suggesting a maternal cancer is uncommon. Furthermore, certain maternal conditions, like vitamin B12 or folate deficiency or autoimmune diseases, or placental mosaicisms may confound the interpretation of NIPT results with regards to the presence of a maternal cancer.

It is evident that identification of a NIPT suggesting a maternal cancer raises a complex and medically, ethically and psychologically challenging situation where the best management options for the mother should be balanced against safeguarding fetal health. Therefore, general awareness and proper understanding about the etiologies of aberrant NIPT outcomes among caregiving physicians, including genetic counsellors, obstetricians and oncologists, is indispensable for prompt and accurate downstream management of NIPT findings that point to the presence of an occult maternal malignancy. Here, we present an overview of the types of NIPT findings being associated with an occult malignancy, latest evolvements in the field as well as proposed recommendations on clinical follow-up of these findings.

## Routine NIPT findings pointing to an occult maternal malignancy

### NIPT results showing multiple CNAs are highly suggestive of an occult maternal malignancy

**Figure 1:**
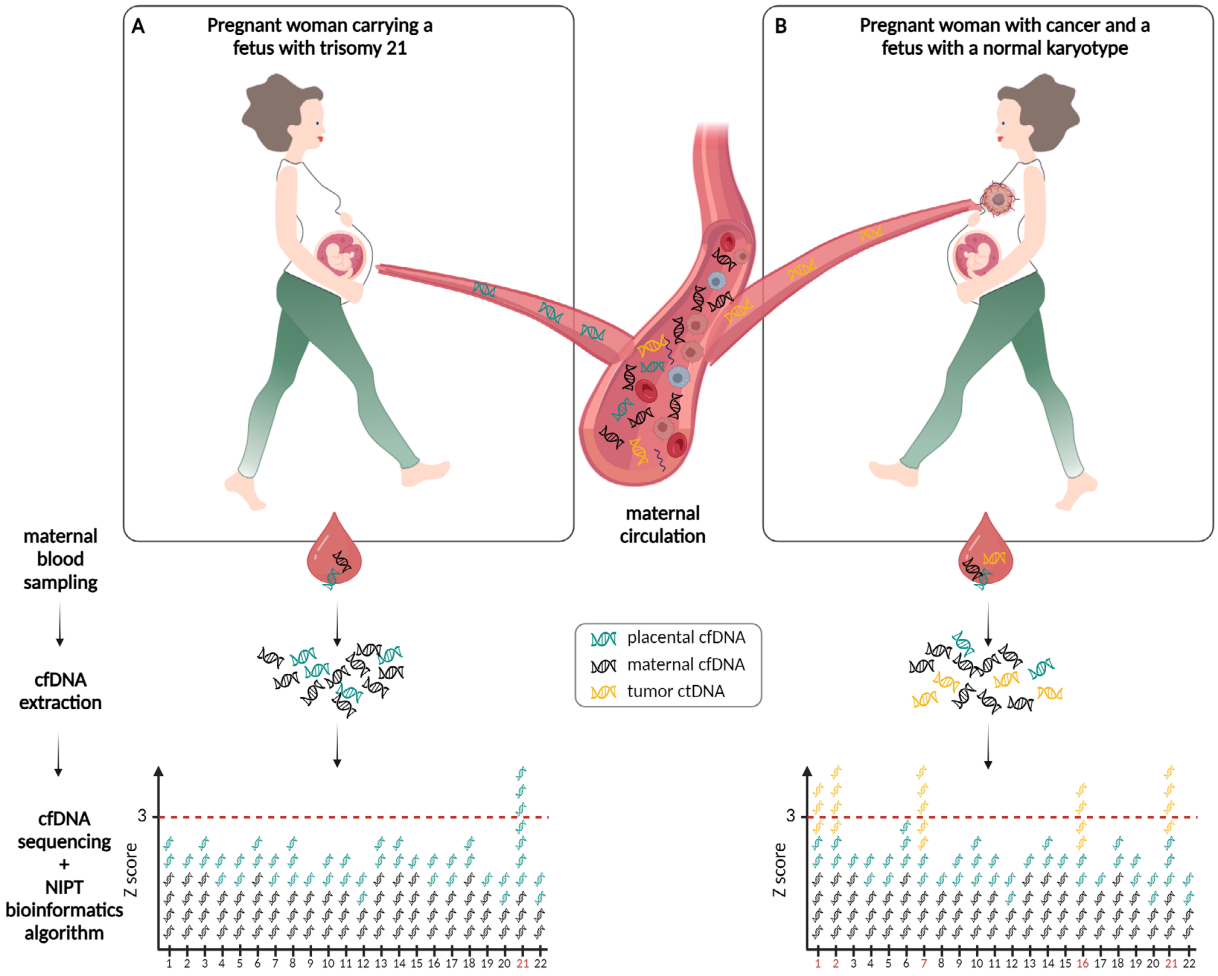
**Detection of fetal-derived and tumor-derived copy number alterations via non-invasive prenatal testing (NIPT) during pregnancy. (A)** Pregnant woman carrying a fetus with trisomy 21. During pregnancy, cell-free DNA (cfDNA) fragments are released from the placenta (green cfDNA strands) into the maternal circulation and mixed with cfDNA from maternal origin (black cfDNA strands). Upon extraction of cfDNA fragments from maternal plasma, genome-wide sequencing of these fragments and subsequent bioinformatics analysis, the presence of a fetal trisomy 21 is visible as an overrepresentation of cfDNA fragments aligning to chromosome 21 (threshold of standard deviation > 3 (z-score) indicated as dotted red line). (**B)** Pregnant woman with an (occult) maternal malignancy and carrying a fetus with a normal karyotype. Cell-free tumor DNA (ctDNA) fragments (yellow ctDNA strands), that represent the genetic makeup of the malignancy including chromosomal aberrations, can be shed in the maternal bloodstream. Upon genome-wide NIPT assessment for the presence of chromosomal aneuploidies in the fetus, tumor-derived CNAs will skew the signals, giving rise to an aberrant NIPT result (elevated z-scores for chromosomes 1, 2, 7, 16 and 21 (chromosome numbers indicated in red on the x-axis); vice versa, in case of a (partial) monosomy, the chromosomal z-score would drop below < –3, not depicted here). When the observed aberrations are characteristic for tumor-derived chromosomal imbalances [22] and incompatible with fetal development, a maternal malignancy might be invoked.

NIPT relies on laboratory and computational analysis of plasma cfDNA to infer the presence of fetal chromosomal aneuploidies [3]. CNAs, comprising segmental and/or whole chromosome aneuploidies, are also a hallmark of cancer [10]. Approximately 90 % of tumors have gained or lost at least one chromosome (arm). As cancer cells have higher turnover rates, and possibly altered cfDNA release mechanisms, they can also shed ctDNA species into the blood circulatory system. Given that tumor-derived chromosomal alterations are retained in these ctDNA fragments, it is plausible that NIPT technologies can identify cancer‐derived CNAs in pregnant women having cancer (Figure 1). Since the first report by Osborne *et al*., linking a postpartum vaginal small cell carcinoma to previously unexplained CNAs in the patient’s NIPT result [11], various reports have confirmed that discordant NIPT findings can be indicative of an occult malignancy in pregnant women [12]–[17]. In particular, the identification of a maternal malignancy was most often associated with the detection of more than one aneuploidy; these complex chromosome arrangements being a well-known hallmark of cancer genomes [11]–[18]. Whereas targeted NIPT assays are limited to their region of interest (most often only interrogating chromosomes 21, 18 and 13), genome‐wide analyses increase the potential to detect such multiple tumor-derived CNAs across the genome. Therefore, when targeted NIPT assays reveal a single autosomal monosomy or trisomy, which would be incompatible with a viable fetus, a more detailed analysis of the whole genome is warranted to determine if a pattern suggestive of malignancy is present [13], [18]. Whole-genome NIPT analysis pipelines also have recurrently discovered the presence of a maternal mosaicism for a single trisomy 8 or a subchromosomal 5q or 20q deletion [14], [19]. Though these chromosomal aberrations are known to be associated with hematologic malignancies, neoplasia’s were not detected in the affected women despite extensive investigations. As the clinical impact of these findings is still unclear, long-term follow-up studies are needed to look into their possible association with neoplasms. Lastly, reports on the identification of a maternal malignancy upon single-nucleotide polymorphism (SNP)-based targeted NIPT technologies are more rare [17]. Yet, given that this method allows distinguishing between maternal and fetal CNAs, it may result in a higher likelihood of detecting a maternal malignancy once a NIPT is found to be suspicious of cancer, as suggested by Goldring *et al.* [17].

Whole-genome sequencing analyses of matched liquid biopsies and tumor samples from series of pregnant cancer patients have confirmed that tumor-specific CNAs in ctDNA were indeed the source of the deviating NIPT findings and have given more insight into the type of chromosomal aberrations that can be expected in NIPT findings in this pregnant cancer patient population [12], [19], [20]. Given that the presence of tumor-derived CNAs can skew the readout of chromosomes 21, 18, and 13 in a NIPT profile, a maternal malignancy may also impede an accurate assessment of the fetal karyotype [21].

**Figure 2:**
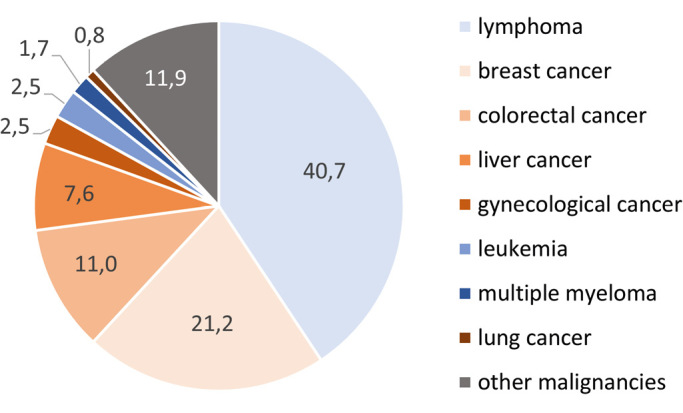
** Frequencies (%) of maternal malignancy types, inferred from NIPT.** Numbers are retrieved from the large, population-based retrospective series on NIPT and detection of a maternal malignancy mentioned in Table 1.

Based on large, population-based retrospective studies, it has been shown that the most frequently identified cancer types are lymphoma’s, followed by breast cancer (Table 1 and Figure 2). Whereas breast cancer is the leading cancer type in females of reproductive age [9], [23], the preponderance of hematological diagnoses via NIPT, such as lymphoma and leukemia (representing almost half of all identified cancer cases), is plausible given the close contact between blood cancer cells and the maternal circulation and the fact that the largest fraction of plasma cfDNA is of hematological origin [24]. Furthermore, differences across countries in the frequencies of cancer types incidentally identified via NIPT may reflect different cancer incidences in the respective general populations (https://canceratlas.cancer.org/the-burden/geographic-diversity/).

It should be noted that also benign tumors, like uterine leiomyomas, can shed ctDNA into the bloodstream which can be detected by NIPT [15]. A prospective study in 13.184 patients undergoing NIPT, of whom 7,7 % had fibroids, showed that these were associated with a low absolute false-positive rate (2 %) for sub-chromosomal aberration del(7)(q22q32), being the most common genetic anomaly in fibroids. Yet, the NIPT screening accuracy for the common autosomal trisomies and sex-chromosomal abnormalities was not altered [25]. This can be explained by the fact that fibroids commonly harbor balanced translocations which do not alter the z-score and hence remain undetected during NIPT assessment. Finally, also vitamin B12 or folate deficiency or the presence of an autoimmune disease like systemic lupus erythematosus may cause abnormal genome-wide cfDNA patterns that can confound NIPT results [26], [27], though the signature of these chromosomal aberrations might be discriminated from typical cancer-like CNAs.

### Cancer detection rates depend on tumor biology, NIPT technology and diagnostic care organization

In large retrospective series, the reported frequencies of an incidental maternal cancer detection via NIPT were in the range of 1 in 8.000 to 1 in 100.000 pregnancies (Table 1). Given that the incidence of pregnancy-associated cancers is estimated to be about 1 in 1.000 to 2.000 [9], it is obvious that a significant number of cancer cases is missed.

First, tumor features, such as cancer type and burden, cellular turnover and accessibility to the circulation, determine ctDNA levels in the bloodstream and hence affect the sensitivity of tumor detection. For instance, due to the blood-brain barrier, patients with tumors located in the central nervous system have lower ctDNA levels in their bloodstream relative to patients with extra-cranial cancer types [28]. Variation also exists among tumors withing the same cancer type, but displaying a different tumor histology. For example, when analyzing plasma cfDNA from pregnant women with a known breast cancer diagnosis using a genome-wide NIPT assay, we found that triple negative breast cancers were more frequently identified compared to hormone-positive or HER2-enriched tumors, potentially due to the presence of high-level gains and losses of cfDNA or high ctDNA loads in plasma of patients with the former tumors [29]. Plasma ctDNA concentrations have also been shown to be correlated with tumor size and stage. Whereas advanced-stage tumors release concentrations of ctDNA than can exceed 10 % of the plasma cfDNA pool [28], limited ctDNA fractions in premalignant and early tumor stages may restrict the sensitivity of detecting cancer in pregnant women [29].

Second, present NIPT technologies are restricted to detect copy variable tumors. As such, they will miss tumors harboring CNAs which sizes are below the detection limit of the NIPT technology used or copy number neutral loss of heterozygosity and, particularly in case of targeted assays, have limited potential to detect single chromosomal aneuploid tumors.

Third, cancer detection rates not only depend on the applied NIPT technology, but also on the efficiency of downstream diagnostic investigations. Amongst published large-scale retrospective studies on the association between NIPT and incidental maternal cancer detection, genome-wide scrutinization of NIPT profiles combined with standardized and comprehensive downstream clinical investigations, resulted in the highest positive predictive value (PPV) reported so far ([19], Table 1). In particular, in the approach presented by Lenaerts *et al*., an extensive diagnostic workup was adopted, including physical examinations, blood work and whole-body magnetic resonance imaging (WB-DWI MRI), thereby applying a low threshold for detecting incidental findings, favouring high sensitivity for lesion detection over specificity [19]. At the same time, multiple investigators reported that a substantial number of suspicious NIPT findings could not be confirmed by subsequent diagnostic tests or physical examinations (30 % up to 90 % of cases, see Table 1). In some of these cases, an occult malignancy (or benign proliferation) may have been below the detection level of current diagnostic techniques. In this regard, there are reports of cases in which chromosomal abnormalities have been observed in NIPT results months to years before clinical symptoms arose [17], [19].

## NIPT and cancer management: current research efforts

From the above, it is clear that NIPT should not (yet) be considered as a cancer screening test. Published reports on NIPT and cancer detection so far were only retrospective in nature. PPVs, extracted from these series, ranged from 8 % to 73 %, depending on the applied technology and diagnostic workup (Table 1). As a consequence, at present, there exist no long-term follow-up data in women with a normal NIPT nor in women with a NIPT finding that was suspicious of cancer yet without a subsequent cancer diagnosis. Hence, the exact number of true and false negatives and positives and thus, the overall sensitivity and specificity of NIPT technologies for detecting different cancer types remains unknown. Furthermore, as from most published reports, detailed oncological information is missing, it is not known whether NIPT can lead to cancer detection at earlier stages and/or whether this would lead to improved patient outcomes. Two prospective studies, i. e. the NIH-funded IDENTIFY study in the United States [30] and a EU4Health-funded European study [31], are currently addressing these knowledge gaps.

**Table 1:** Population-based retrospective series investigating the association between discordant NIPT results and maternal malignancies

								**type of reported maternal malignancies**															
								**solid cancer type**						**hematological cancer type**									
**Reference**		**type of test**	**number of NIPT tests**	**NIPT tests suspicious of maternal cancer**	**malignancies reported (in those with follow-up)**	**detection rate of cancers in pregnancy**	**PPV (%)**	**time NIPT to cancer diagnosis (days)**	**breast cancer**	**cervical cancer**	**ovarian cancer**	**liver cancer**	**colorectal cancer**	**lung cancer**	**lymphoma, type not specified**	**Hodgkin lymphoma**	**non-Hodgkin lymphoma**	**follicular lymphoma**	**T-cell lymphoma**	**multiple myeloma**	**chronic myeloid leukemia**	**acute myeloid leukemia**	**other malignancies or not specified**	
Goldring *et al*.	[17]	SNP–based technology, targeted analysis	2.004.428	38	20 (30)	1 : 100.221	66,7	0–330 (median 210)	5		1		3		6	4							1	
Heesterbeek *et al*.	[14]	sequencing, genome-wide analysis	168.452	48	16 (48)	1 : 10.528	33,3	567 or less (median 35)	4				1			7	4					1	1	
		sequencing, targeted analysis	63.444	3	2 (3)	1 : 31.722	66,7															
Lenaerts *et al*.	[19]	sequencing, genome-wide analysis	88.294	15	11 (15)	1 : 8.027	73,3	6–57 (median 32)	2	1						3	3			1		1	
Ji *et al*.	[16]	sequencing, genome-wide analysis	1.930.000	639	41 (542)	1 : 47.073	7,6	0–366 (median 115)	10	1		9	7	1	9								4	
Dharajiya *et al*.	[15]	sequencing, targeted analysis	450.000	55	18 (43)	1 : 25.000	41,9	na	4				1			4	1	1	1	1	1		4	
Bianchi *et al*.	[13]	sequencing, targeted analysis	125.426	na	10 (na)	1 : 12.543	na	21 – 273 (median 84)					1			1	3		1				4	

na, not available; PPV, positive predictive value; SNP, single nucleotide polymorphism

Given that NIPT has provided proof-of-principle of a liquid biopsy test to detect neoplasia, we and others explored the use of this technology for cancer screening and management in nonpregnant populations. Cohen *et al*. applied whole-genome NIPT analyses on preoperative plasma samples from nonpregnant patients with a known high-grade serous ovarian carcinoma and showed that 41 % of all cancers, including 38 % of early stage cases, could be detected [32]. When applying GIPSeq, an *in house* developed genome-wide NIPT pipeline, on plasma cfDNA samples of 1.002 elderly men and women without a prior malignancy, we identified 5 incipient hematological malignancies and 1 myelodysplastic syndrome [33], again underscoring the potential of NIPT as an unbiased screening approach for hematological malignancies and premalignant conditions such as clonal hematopoiesis of indetermined potential. Furthermore, low coverage whole-genome sequencing analysis used for NIPT was shown to be able to identify CNAs in ctDNA of ovarian [34] and breast [29] cancer patients before and after chemotherapy, highlighting its potential application for treatment monitoring.

In the past years, much progress has been made in the development of non-invasive multi-cancer early detection (MCED) tests intended to simultaneously screen for multiple cancer types [35]. These MCED tests rely on the analysis of circulating proteins, genetic (mutations, CNAs, …) or epigenetic (methylation, fragmentation, …) features of ctDNA or a combination of these biomarkers, thereby using machine learning methods for analyzing these complex datasets. In a comparative evaluation of MCED tests, those using cfDNA methylation markers had higher cancer signal detection sensitivities than tests based on somatic CNA detection [36]. It is thus likely that these MCED tests may be superior to NIPT for early cancer detection. Yet, similar as mentioned above for NIPT, many unknowns remain to be addressed, such as the risks of false positives and negatives, the extent of the downstream diagnostic workup when the MCED test suggests an occult malignancy, or clinical utility endpoints like patient cancer-related mortality [37], [38].

## Management recommendations for NIPT findings suggestive of a maternal cancer

### NIPT findings pointing to a maternal malignancy are actionable

Reporting the presence of a potential cancer during pregnancy is inevitably a balance between the value of diagnosing a true cancer and the risk of a false positive causing unnecessary stress about fetal and maternal outcomes and invasive follow-up testing. If high PPVs can be achieved (by improving specificity while maintaining a high sensitivity), the value of reporting outweighs the associated risks. This is particularly true since a cancer diagnosis during pregnancy can be actionable. Indeed, management options for patients with cancer during pregnancy are expanding and certain anti-cancer therapies are relatively safe if given during the second or third trimester of pregnancy, without major adverse effects on short-term pediatric outcome [39]. Conversely, early chemotherapy treatment has been shown to enable a reduction in pregnancy terminations and premature births and may offer a similar maternal prognosis to that of nonpregnant women [40]–[42]. Moreover, diagnosing a malignancy during pregnancy also allows early identification of possible obstetric risks.

### Proposed recommendations for NIPT findings suggesting an incipient maternal malignancy

As prospective data are lacking, there are currently no evidence-based guidelines for clinical follow-up when a NIPT finding suggests an occult maternal malignancy. For example, Belgian guidelines specify which maternal incidental findings (including maternal malignancies) should be reported, yet no direction is given on the clinical management of these findings [43]. In the past years, a number of clinical care paths have being proposed for the management of NIPT findings that are suspicious of a maternal malignancy [19], [44]–[46]. Central in these propositions is the call for a multidisciplinary approach, involving caregivers from the genetics, oncology and obstetric units. Given the complexity of handling a cancer diagnosis during pregnancy, this crosstalk is deemed necessary to outline a diagnostic and therapeutic management, taking into account both maternal and fetal health.

**Figure 3:**
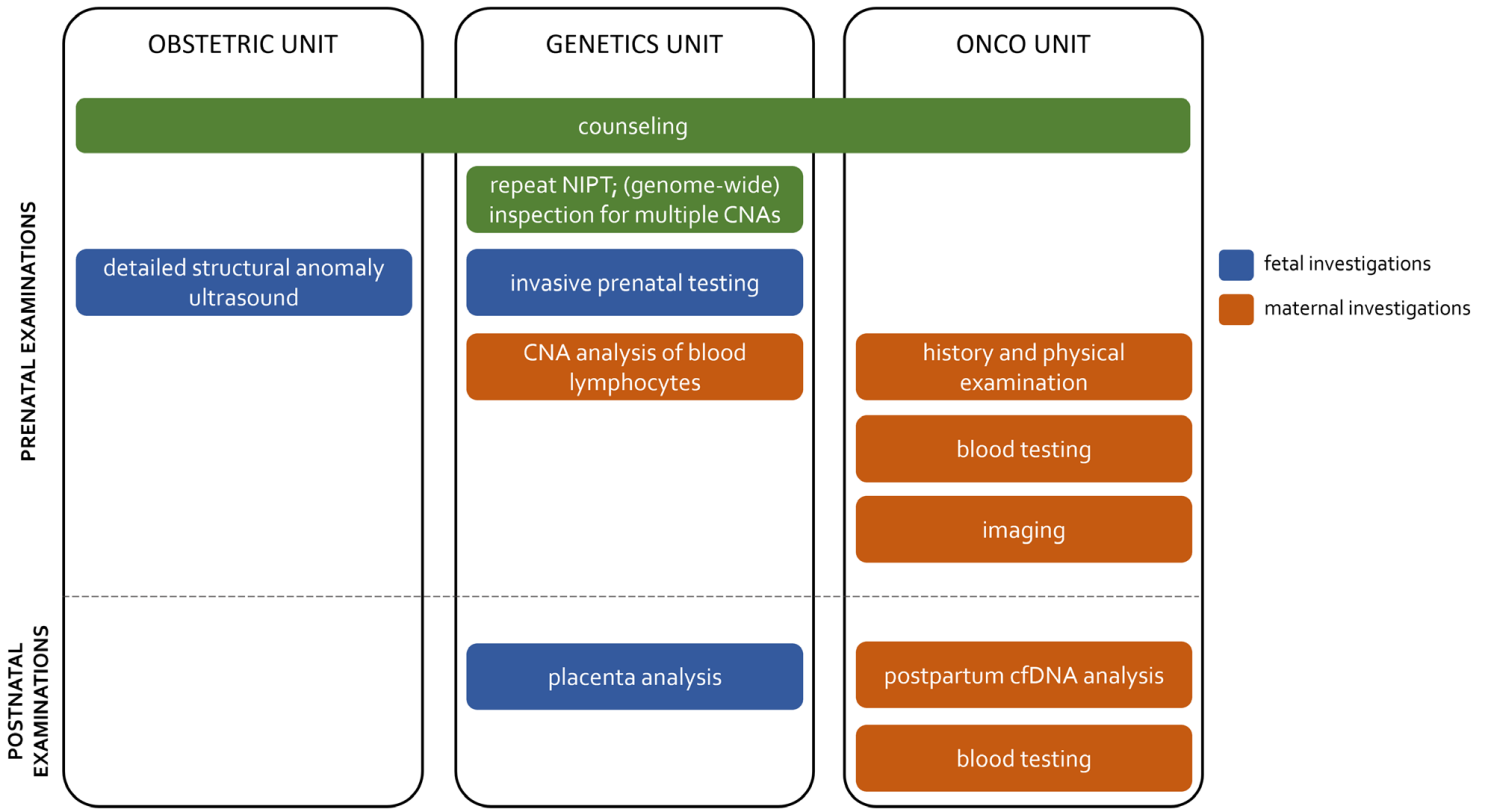
Critical elements in a multidisciplinary management model for downstream clinical investigations in pregnant women confronted with a NIPT that is suggestive of an incipient tumor.

Proposed clinical care paths suggest a stepwise workup, including certain critical elements (Figure 3).

**Genetics laboratory analyses.** In case of an unusual NIPT outcome, a repeat NIPT could be performed on an independent blood sample to exclude technical issues. When the observed chromosomal aberrations are incompatible with normal fetal development, in particular for multiple CNAs or a single full monosomy, the potential presence of an occult maternal malignancy should come to mind. If the laboratory performing NIPT does not initially report genome-wide data underlying an unusual NIPT result, e. g. a single monosomy/trisomy incompatible with a viable fetus in case of targeted NIPT analyses, it may be of benefit to request these data to determine whether multiple CNAs are present.
**Counseling.** The possibility of incidental findings, such as an incipient maternal cancer, should already be included in pre-test counseling discussions. Dependent on local guidelines and socio-cultural factors, families could be offered the option to opt out of being informed about findings beyond the aneuploidy status of the fetal chromosomes. Proper post-test counselling by a clinical geneticist should also be in place. Lannoo and colleagues provided a comprehensive overview of answers to questions of pregnant women and caretakers confronted with a NIPT suggesting a maternal malignancy [46]. Amongst others, the possibility that benign tumors, such as uterine leiomyomas, or systemic maternal diseases can also cause aberrant CNA profiles, should be mentioned.
**Fetal investigations**. When a maternal cancer is suspected, the presence of large ctDNA fractions in maternal blood may mask the fetal chromosomal profile and prevent a reliable estimation of the risk of fetal trisomy 21, 18 and 13 [21]. Therefore, the patient should be referred to a perinatologist-obstetrician with experience in invasive prenatal diagnostics.
  Detailed structural anomaly screening should be done via ultrasound.
  Though NIPT profiles with multiple large CNAs are considered incompatible with normal fetal development, the possibility of an invasive prenatal test (amniocentesis) can be offered if certainty on fetal chromosomal abnormalities is desired. The risks associated with the invasive test should be weighed against the anxiety of the expecting parents and the risk of an abnormal fetal karyotype. In our experience, pregnant women with a putative cancer diagnosis generally opted for invasive prenatal testing.
**Maternal investigations.** When the results are suggestive of an underlying maternal disorder, the patient should be referred to a multidisciplinary team consisting of a medical and gynecological oncologist, hematologist and imaging specialists, to initiate maternal diagnostic investigations.
  Maternal genetic testing on multiple tissues could be performed to interrogate the possibility of specific (constitutional mosaic) chromosomal patterns.
  Comprehensive clinical history and physical examination should be done.
  Since plasma tumor markers on their own show moderate diagnostic sensitivity and are less reliable in pregnancy [47], imaging is indicated to identify the primary tumor site. WB-DWI MRI allows for radiation-free imaging of the entire body during one examination. Alternatively to WB-DWI MRI, sequential organ-specific examinations may be applied [46].
  As, prior to breast cancer, lymphomas are the most frequently detected tumor type following a discordant NIPT outcome, imaging should be complemented with the analysis of hematological parameters (such as cell counts, cytology and clinical biochemistry) to search for the presence of a hematological malignancy that might not be detectable via imaging.
When a malignancy is identified, **management options for pregnant cancer patients** should always be discussed by an expert multidisciplinary team having the resources and experience to manage the complex issues that arise with regard to fetal, obstetrical and maternal outcomes. In this regard, physicians seeking recommendations on the management of pregnant cancer patients, can now rely on a virtual multidisciplinary tumor board giving on-demand and free-of-charge advice on this topic, without boundaries of hospitals or countries (The Advisory Board on Cancer, Infertility and Pregnancy, ABCIP, www.ab-cip.org [48]).
Would the oncological investigations be negative, **postpartum evaluation** of a placental biopsy will allow examining the possibility of NIPT findings originating from confined placental mosaicism, such as trisomy 8. Likewise, a postpartum liquid biopsy could be performed to evaluate the evolution of the cfDNA CNA profile. Technically, commercially available MCED tests could also be used for this purpose. Yet, these tests are not yet approved by medicines regulatory authorities and, as mentioned before, there is little clarity on the best practices for use for cancer screening nor has their clinical utility been demonstrated yet. Finally, yearly clinical assessment and blood tests for hematological parameters could be considered. In individuals with detectable clonal mosaicism for large chromosomal abnormalities in peripheral blood cells, a 10-fold increased risk of future hematologic cancer diagnosis has been seen [49]. As mentioned above, there exist reports of patients that have being diagnosed with cancer up to 3 years after a suspicious NIPT finding and an initial negative cancer workup [17], [19].

## Advancements in the liquid biopsy field affecting NIPT

At present, when a NIPT finding is suggestive of an incipient tumor, it is difficult to pinpoint where a putative tumor may be located, solely based on the analysis of genomic aberrations. The group of *Li* and colleagues combined NIPT data with plasma tumor protein markers to develop a classifier for the prediction of breast or liver cancer or lymphoma [50]. However, it can be questioned whether this approach is generalizable towards identification of other tumor types in pregnant women given that frequently used circulating tumor protein markers (like CA 125, squamous cell carcinoma antigen and CA 15.3) may be unreliable due to pregnancy-specific variations and result in false positive signals [47].

Within the liquid biopsy field, increased understanding of the biology and physical nature of cfDNA has stimulated the development of approaches to identify the tissue-of-origin of cfDNAs. In particular, the analysis of epigenetic features of cfDNA molecules, such as their fragmentation pattern or methylation status, together with advancements in computational methods, have been shown to allow estimating the proportions of different tissue and cell types in cfDNA mixtures [51], [52]. Certain MCED tests have included this feature to support downstream diagnostic confirmation of the tissue-of-origin in patients who screen positive in the pan-cancer detection tests [35]. At present, efforts to integrate multiple datasets of cfDNA features (e. g. genomics, fragmentomics, nucleosomics and methylomics) in so-called ‘multi-view approaches’ using innovative machine learning algorithms are expected to further improve the performance characteristics (such as sensitivity, specificity and identification of the tissue-of-origin of unexplained cfDNA signals) of liquid biopsy tests [52]. It can be anticipated that these advancements may also find their way to clinical prenatal testing and can contribute to distinguishing fetal from maternal cfDNA molecules and inform about the origin of cancerous tissues. This would allow organ-targeted imaging approaches and thus prevent a diagnostic odyssey.

## Conclusion

When identifying discordant NIPT findings, and in particular when these are characterized by multiple CNAs, a potential maternal cancer should come to mind. At present, only retrospective evaluations have been published, thereby making use of different methods and analysis pipelines and thus preventing an accurate deduction of the frequency of these findings. It is apparent that identification of an incipient maternal malignancy is dependent on efficient downstream clinical follow investigations. Yet, currently no standardized workup guidelines exist for such NIPT findings that are suspicious of a maternal cancer. Whereas future analytical and computational advancements in the liquid biopsy field may also be translated to prenatal testing and are anticipated to further improve NIPT prediction of maternal neoplasia, ongoing large-scale prospective evaluations on NIPT and cancer detection are expected to provide evidence on the best clinical practices and on whether identification of a cancer via NIPT leads to improved patient outcomes. Meanwhile, a clinical management scheme in a multidisciplinary expert setting is advocated for NIPT outcomes suggesting an occult maternal malignancy.
